# Time-series analysis of ruminant foetal wastage at a slaughterhouse in North Central Nigeria between 2001 and 2012

**DOI:** 10.4102/ojvr.v82i1.1010

**Published:** 2015-12-15

**Authors:** Nma B. Alhaji, Ismail A. Odetokun, Aminu Shittu, Joshua Onyango, Umar M. Chafe, Muhammed S. Abubakar, Issa A. Muraina, Folorunso O. Fasina, Hu Suk Lee

**Affiliations:** 1 Epidemiology Unit, Ministry of Livestock and Fisheries Development, Nigeria; 2 Department of Public Health and Preventive Medicine, University of Ilorin, Nigeria; 3 Department of Theriogenology and Animal Production, Usmanu Danfodiyo University, Nigeria; 4 Animal Welfare and Veterinary Health, University of Northampton, United Kingdom; 5 Department of Medicine and Surgery, Usmanu Danfodiyo University, Nigeria; 6 Department of Pathology and Microbiology, Usmanu Danfodiyo University, Nigeria; 7 Diabetes and Nutritional Science Division, King’s College, United Kingdom; 8 National Veterinary Research Institute, Akure, Nigeria; 9 Department of Production Animal Studies, University of Pretoria, South Africa; 10 International Livestock Research Institute, Regional Office for East and Southeast Asia, Hanoi, Vietnam

## Abstract

In developing countries, foetal wastage from slaughtered ruminants and the associated economic losses appear to be substantial. However, only a limited number of studies have comprehensively evaluated these trends. In the current study, secondary (retrospective) and primary data were collected and evaluated to estimate the prevalence of foetal wastage from cattle, sheep and goats slaughtered at an abattoir in Minna, Nigeria, over a 12-year period (January 2001 – December 2012). Time-series modelling revealed substantial differences in the rate of foetal wastage amongst the slaughtered species, with more lambs having been wasted than calves or kids. Seasonal effects seem to influence rates of foetal wastage and certain months in the year appear to be associated with higher odds of foetal wastage. Improved management systems are suggested to reduce the risk of foetal losses.

## Introduction

Ruminant livestock (cattle, sheep and goats) are a source of food (meat and milk) and are also used to produce animal feed, clothing, shelter, weapons, utensils and industrial items (Church 1993). Food animals also play an important role in providing financial security for rural populations and in improving a nation’s economy, especially in developing countries (Alhaji 2011; Alhaji & Odetokun 2013). In Nigeria, the annual consumption of animal protein (meat, milk, eggs and fish) amounts to 8.8 kg/person compared to the global average of 41.9 kg/person (Food and Agriculture Organization [FAO] 2013). Countries such as Australia and the USA consume approximately 111.5 kg/person/year and 120.2 kg/person/year, respectively (FAO 2013). The comparison suggests that Nigeria still rates as a developing country, with low per capita production and consumption of animal protein (Nwakpu & Ugwu 2004).

Nigeria’s cattle, sheep and goat populations were recently estimated at 19.2 million, 38.5 million and 57.4 million, respectively (FAO 2014), with annual growth rates that are too low to satisfy the requirements of the increasing human population. The Nigerian population was estimated at over 174 million in 2013 (Population Reference Bureau 2013) and a relative increase in wealth will mean that the demand for meat as a source of protein will continue to grow. The high demand for sources of animal protein has led to practices such as indiscriminate slaughter of young and pregnant female animals, which is seen routinely in some Nigerian abattoirs (Taiwo, Aluko & Olufowobi 2006). This practice results in wastage of scarce protein available to consumers and a decrease in the livestock growth capacity of the country owing to low herd replacement rates (Cadmus & Adesokan 2010).

The indiscriminate slaughter of pregnant females and the consequent wastage of embryos and foetuses are regarded as major destructive mechanisms that counteract food production efforts (Abassa 1995). The magnitude of foetal losses due to the slaughter of reproductively active dams has been reported amongst both large (cows) and small (ewes and does) ruminants from several abattoirs in the South West (Cadmus & Adesokan 2010; Oyekunle, Olubanjo & Fasina 1992), South East (Wosu 1988; Wosu & Dibua 1992), North Central (Alhaji 2011; Alhaji & Odetokun 2013), North West (Garba *et al*. 2010; Muhammad, Ashiru & Abdullahi 2007) and North East (Bokko 2011; Chaudhari & Bokko 2000) zones of Nigeria.

The practice of slaughtering pregnant ruminants has had a negative effect on the national herd size and total meat production, restricting availability of animal protein. The associated economic losses constrain the contribution of livestock to the gross domestic product in the country (Alhaji 2011; Alhaji & Odetokun 2013; Ngbede *et al.* 2012). Substantial foetal calf wastage has been recorded at an abattoir in northern Nigeria (Alhaji 2011), which translated to an average annual financial loss of close to US$290 000 (Ngbede *et al*. 2012). Reasons for the reported continuous slaughter of pregnant ruminants should be investigated to predict associated trends and facilitate sound strategic planning and decision-making in combating future foetal livestock wastage in Nigeria.

None of the aforementioned reports have adequately addressed an empirical trend analysis of ruminant foetal wastage. Specifically, seasonal slaughter trends should be studied to determine slaughter patterns to allow future predictions. Previous limitations in this regard may be due to a lack of detailed abattoir slaughter records, only short periods covered by available information and incomplete data. We present an empirically sound trend analysis to evaluate and compare seasonal patterns and variations of ruminant foetal wastage in Nigeria over a 12-year period.

## Materials and methods

### Study area

The study was conducted at the Minna Metropolitan Abattoir (MMA), a major slaughterhouse in the capital of Niger State, North Central Nigeria (6°1′N; 9°5′E) (see Figure 1). In 2007, the human population in the city was estimated at just more than 304 000.

**FIGURE 1:**
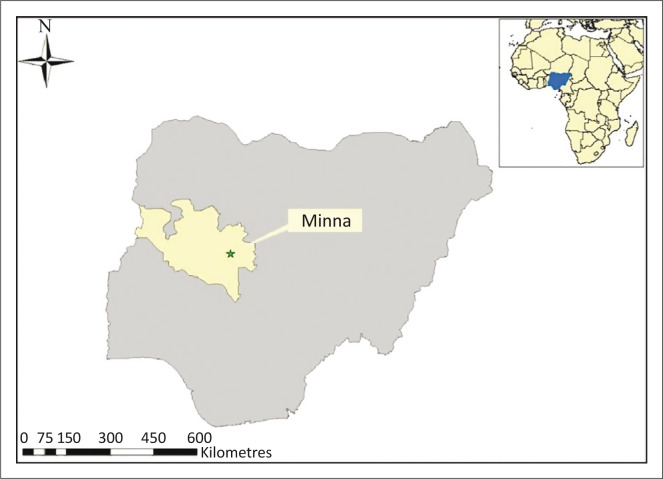
Map of Nigeria, with the location of Minna in the northern central region of Nigeria indicated.

### Data sources

Secondary data on animals presented at the abattoir between 2001 and 2010 were obtained retrospectively from the records of the Zonal Veterinary Office, Niger State Ministry of Livestock and Fisheries Development, Minna. In addition, primary data were obtained for 2011 and 2012 from direct ante- and post-mortem inspections in the lairage and slaughterhouse of the MMA. The retrospective data were considered valid based on the detailed pregnancy statuses and foetal wastages recorded. All the female animals presented for slaughter between 2011 and 2012 were assessed for pregnancy status and foetal wastage at the slaughterhouse and the data recorded. The data were entered into a spreadsheet and exported to statistical software applications (Stata version 11.2 [StataCorp, College Station] and R version 3.2.2 [https://cran.r-project.org/bin/windows/base/]). Abattoir foetal wastage was defined as the removal and subsequent discard of one or more foetuses from the gravid uterus of a female animal.

### Seasonal-trend decomposition and statistical analyses

Monthly and total foetal wastage rates were expressed as the total number of pregnant females (*n*_preg_) divided by the total number of female animals slaughtered (*N*_preg + not-preg_). Boxplots were generated to display the upper and lower quartiles of groups of numerical data, with the whiskers indicating variability outside these parameters. Foetal wastage, throughputs and slaughter rates of pregnant animals were analysed by month and by season across all animals combined and for individual species. A seasonal-trend decomposition procedure based on a locally weighted regression (STL) (Cleveland *et al*. 1990) was used for the time-series analyses. Months were used as the data time units for seasonality and trend. STL is a useful technique to visualise time-series data sets in which periods can be decomposed into three separate parts: trend, seasonal and remainder components. The STL procedure involves a sequence of applications with locally weighted regression (loess), which identify data patterns that do not conform to the mathematical polynomial equations. Annual patterns were determined as seasonal components over the 12 years, followed by smoothing to determine trends. The remaining components were the monthly residuals from the seasonal and trend fits. Monthly variations in foetal wastage were illustrated using a seasonal cycle subseries plot that assumed yearly periodicity. The seasonal cycle subseries plots display horizontal lines for the mean rate of foetal wastage for each month over the entire study period, whereas the end of vertical lines emanating from the horizontal lines indicate the specific rates of foetal wastage for that month in each year of the study.

Based on the seasonal cycle subseries plot, patterns between and within groups can be visualised for the total study period. The plot basically shows the average foetal wastage for each month (January–December) of the study period plotted as a horizontal line. The vertical lines drawn from each horizontal line display the individual patterns for the relevant month in each year. However, the seasonal cycle subseries plots were interpreted with caution, as the horizontal lines (average for vertical lines) were substantially affected by large values. As the STL cycle subseries plots do not provide any statistical comparisons, we used logistic regression analysis for additional analyses of the monthly or seasonal differences, as applied elsewhere (Lee *et al*. 2014). The univariable logistic regression model was employed for each animal with an odds ratio and 95% confidence interval (CI) for each month to compare average monthly foetal wastage statistically. The month with the lowest values of foetal wastage (November) was used as a reference month. Differences were considered statistically significant at *P* < 0.05.

## Results

In the period under review, a total of 536 405 animals were slaughtered at the MMA, of which 260 849 were cattle, 8051 sheep and 267 505 goats (Table 1). A total of 9948 pregnant animals were slaughtered, representing 4.02% (95% CI: 3.94–4.10) of the total number of female animals slaughtered during 2001–2012. However, proportionately more pregnant sheep (15.89%; 95% CI: 14.80–17.03) were slaughtered than cattle (4.53%; 95% CI: 4.42–4.64) or goats (2.99%; 95% CI: 2.89–3.09). Also, more male (53.83%; 95% CI: 53.69–53.96) than female animals (46.17%; 95% CI: 46.04–46.31) were slaughtered (Table 1). In total, foetuses of 5929 calves, 755 lambs and 4740 kids were wasted (Table 1).

**TABLE 1:** Summary of the number of ruminants slaughtered at Minna Municipal Abattoir, Nigeria, 2001–2012.

Animal species	Animals slaughtered		Sex				Pregnant female animals (*n*)	Wasted foetuses (*n*)	Foetal wastage (%); CI_95%_
	Number	Proportion of total (%); CI_95%_	Male	Proportion of total (%); CI_95%_	Female	Proportion of total (%); CI_95%_			
Cattle	260 849	48.63; 48.50–48.76	129 891	49.80; 49.60–49.99	130 958	50.20; 50.01–50.40	5929	5929	4.53; 4.42–4.64
Sheep	8051	1.50 1.47–1.53	3930	48.81; 47.72–49.91	4121	51.19; 50.09–52.28	655	755	15.89; 14.80–17.03
Goats	267 505	49.87; 49.74–50.00	154 914	57.91; 57.72–58.10	112 591	42.09; 41.90–42.28	3364	4740	2.99; 2.89–3.09
**Total**	**536 405**	** **	**288 735**	**53.83; 53.69–53.96**	**247 670**	**46.17; 46.04–46.31**	**9948**	**11 424**	**4.02; 3.94–4.10**

CI_95%_; 95% confidence interval.

Figure 2 shows the mean monthly and seasonal throughputs across all animal species slaughtered. The monthly average throughput was 1242 (Figure 2a), with the lowest throughput obtained in February (995), whereas August produced the highest throughput (1489). Daily throughput was highest (1366) in the late rainy season, with the lowest throughput (1079) reported for the late dry season (Figure 2b). The evaluation of monthly slaughter patterns showed that more pregnant animals were slaughtered in August and early dry to early rainy seasons (Figure 2c, d). Total monthly and seasonal foetal wastage patterns across all species were similar (Figure 2e, f).

**FIGURE 2:**
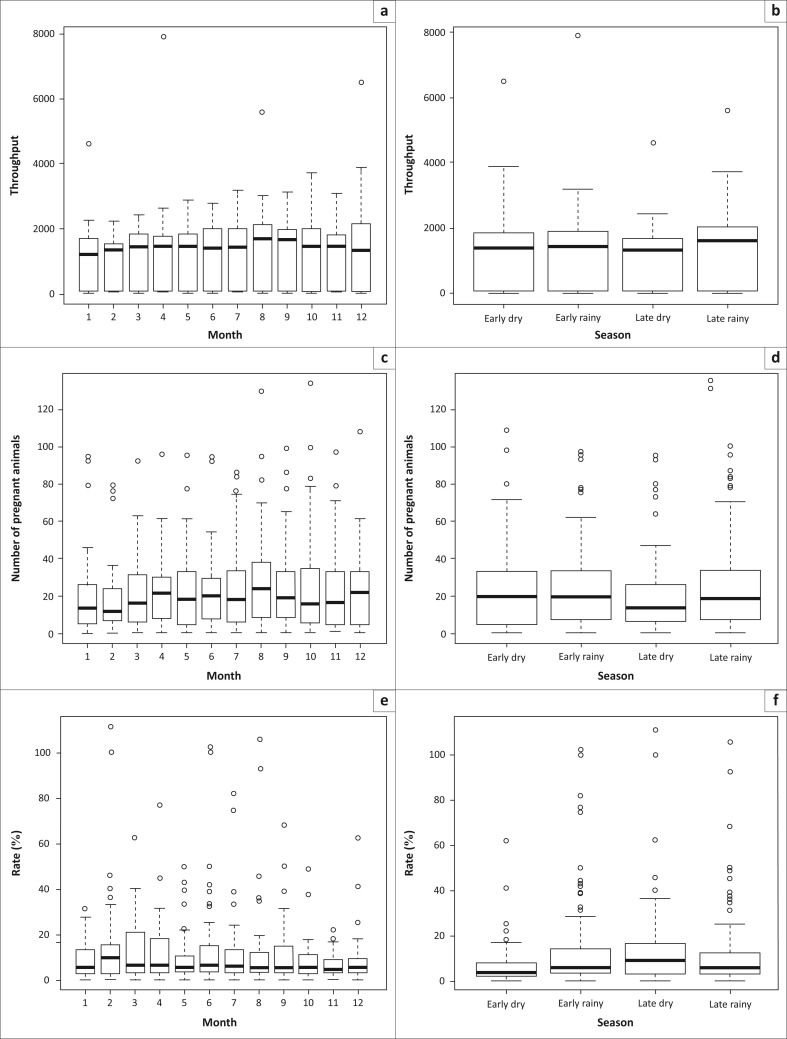
Monthly and seasonal rates of throughput, pregnant animals slaughtered and foetal wastages observed for the period 2001–2012 at the Minna Metropolitan Abattoir, Nigeria. (a) Monthly throughput (all species), (b) seasonal throughput (all species), (c) monthly slaughter (all pregnant animals), (d) seasonal slaughter (all pregnant animals), (e) monthly rate of foetal wastages (all species) and (f) seasonal rate of foetal wastages (all species).

The STL plots (Figure 3) indicate that seasonality appears to be the strongest component in determining slaughter patterns during the study period. For cattle, the STL plot of the trend component (Figure 3a, top row) appears fairly consistent, except for a sharp increase between 2002 and 2003. The seasonal component (Figure 3a, middle row) shows a single peak (≤ 7%) per year, with the highest foetal wastage rates observed from the middle to later months of the year (May–October). The remainder component appears to have random variations (Figure 3a, bottom row), although a large positive residual was noted in October 2002. In addition, the seasonal cycle subseries plot shows that the mean monthly foetal wastage rates were greatest in October (*χ*^2^ = 167.75; *P* < 0.0001) (Figure 4a; Appendix 1 and 2). The cycle subseries plot also shows monthly variations across different years, as indicated by the vertical peaks (Figure 4a). Compared with November as standard, the odds of calf wastage were slightly higher in July, August and October, but lower in February, March and December (Table 2).

**FIGURE 3:**
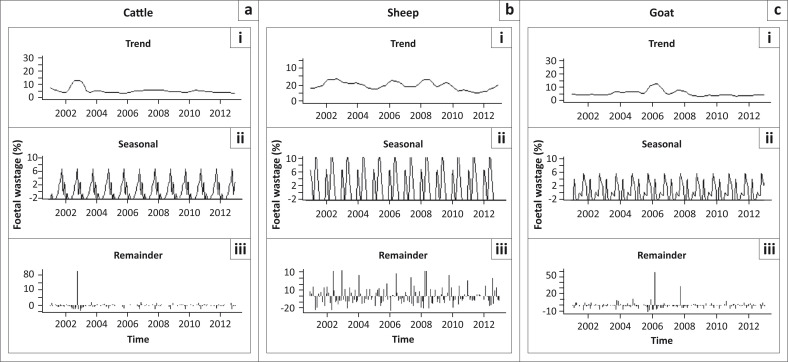
Seasonal-trend decomposition of monthly rates of slaughter and foetal wastage at the Minna Metropolitan Abattoir, Nigeria, for the period 2001–2012. (a) Cattle, (b) Sheep and (c) Goats.

**FIGURE 4:**
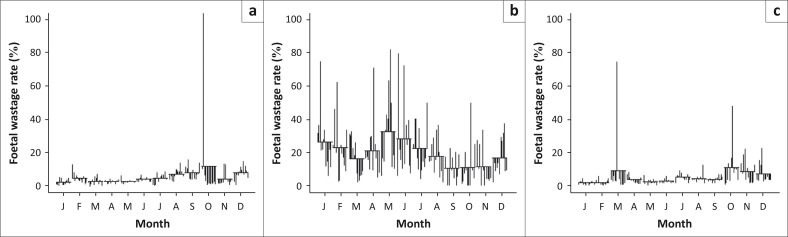
Cycle subseries plot from data of (a) cattle, (b) sheep and (c) goats slaughtered at Minna Metropolitan Abattoir, Nigeria, during the period 2001–2012.

**TABLE 2:** Univariable logistic regression results for foetal wastage rate, expressed as odds ratios, for the period 2001–2012. Data are organised according to month for each animal species.

Month	Cattle (CI_95%_)	Sheep (CI_95%_)	Goats (CI_95%_)
January	1.05 (0.93–1.19)	1.46 (0.93–2.30)	1.02 (0.85–1.24)
February	0.82* (0.72–0.94)	2.00* (1.27–3.16)	1.26* (1.04–1.52)
March	0.85* (0.74–0.97)	2.06* (1.30–3.25)	1.42* (1.20–1.70)
April	1.00 (0.88–1.14)	2.58* (1.65–4.04)	0.79* (0.67–0.94)
May	0.88 (0.78–1.00)	1.82* (1.13–2.95)	1.37* (1.15–1.63)
June	1.08 (0.95–1.22)	1.95* (1.24–3.07)	1.28* (1.08–1.53)
July	1.17* (1.04–1.33)	1.96* (1.19–3.21)	1.12 (0.94–1.34)
August	1.17* (1.04–1.31)	2.35* (1.49–3.71)	0.96 (0.81–1.14)
September	0.97 (0.86–1.10)	2.06* (1.31–3.25)	1.01 (0.85–1.20)
October	1.16* (1.03–1.31)	1.55 (0.97–2.47)	1.14 (0.96–1.36)
November	Reference: 1.00	Reference: 1.00	Reference: 1.00
December	0.65* (0.57–0.73)	1.16 (0.73–1.86)	1.05 (0.88–1.24)

*Statistically significant at *P* < 0.05.

CI_95%_: 95% confidence interval.

For sheep data, the STL plot of the trend component (Figure 3b, top row) shows major fluctuations during the 12-year study period. The seasonal component (Figure 3b, middle row) shows two peaks (> 10%) per year, with the highest foetal wastage rates between December and February, and March and June, respectively. Other months were quiet with regard to foetal wastages. The remainder component (Figure 3b, bottom row) shows random variations, although large positive residuals were noted intermittently throughout the study period. The seasonal cycle subseries plot shows that mean monthly foetal wastage rates were greatest in May and least between September and November (*χ*^2^ = 36.78; *P* = 0.0001) (Figure 4b; Appendix 1 and 2). The odds of lamb wastage were significantly higher between February and September every year (Table 2).

For goats, the STL plot of the trend component shows consistent minor fluctuations over the study period, with a significant peak in 2006 and a lesser one in 2008 (Figure 3c, top row). The seasonal component (Figure 3c, middle row) indicates two peaks (≤ 6%) per year, with the highest foetal wastage rates between August and December, January and March, and May and July. The remaining components also have random variations (Figure 3c, bottom row), although large positive residuals were noted in March 2006 and October 2007. The seasonal cycle subseries plot shows that mean monthly foetal wastage rates were greatest in October, closely followed by a peak in March (*χ*^2^ = 88.17; *P* < 0.0001) (Figure 4c; Appendix 1 and 2). The odds of kid wastage were slightly elevated in February, March, May and June, but lower in April (Table 2).

The odds for foetal wastages varied for individual species and across months, but were generally higher for sheep than for goats or cattle (Table 2).

## Discussion

This study used the STL method to evaluate the trends and seasonal patterns of abattoir slaughter and foetal wastage at a Nigerian abattoir over a 12-year period. Based on significant data sets from slaughtered cattle, sheep and goats, variations with regard to trend, seasonality and remainder components were observed. Seasonality (particularly late rains) affected slaughter patterns and foetal wastage at the MMA between 2001 and 2012 most notably. The results showed that annual trends alone, without considering the effect of other parameters, could not predict the rates of ruminant foetal losses at the studied abattoir. There was no consistent trend observed for cattle, sheep or goats with regard to slaughter and foetal wastage. Slaughter of pregnant ruminants has also been reported from Cameroon (Ndi, Tambi & Agharih 1993), Ethiopia (Mekibib, Desta & Tesfaye 2013), Ghana (Atawalna *et al*. 2013), Mali (Wilson & Traore 1988), Tanzania (Assey *et al*. 1998), Gambia (Goossens *et al*. 1998), Zambia (Zulu *et al*. 2013), Australia (Ladds, Summers & Humphrey 1975) and New Zealand (Lawton, Mead & Baldwin 2000).

In Nigeria, the agricultural year is divided into two major seasons, namely the rainy season (April–October) and the dry season (November–March). Foetal wastage peaked in the dry seasons or immediately afterwards, which coincides roughly with periods when most of the animals lose significant body condition owing to drought-related food scarcity. The sale of extensively raised cattle appears to surge during this period to recover some funds given the sparse feed supply. In addition, as no controlled breeding is observed for these livestock, pregnant animals may be sold off to an abattoir, which subsequently results in foetal wastages. Although previous studies have concluded that annual foetal losses amongst cattle occur more often in the dry seasons in slaughterhouses and abattoirs across Nigeria (Cadmus & Adesokan 2010; Chaudhari & Bokko 2000; Ngbede *et al*. 2012; Nwakpu & Osakwe 2007; Tizhe *et al*. 2010) and Cameroon (Ndi *et al*. 1993), our study shows a similar result also for goats.

However, a different pattern was observed for foetal wastage of sheep, as foetal wastage rates were relatively lower between November and March. It is unclear whether this pattern was due to the relative paucity of data for sheep compared to other species; however, we are aware that festivities notably affect patterns of sheep slaughter in Nigeria. The adoption of feed reservation in stores for use in periods of feed scarcity and the practice of zero grazing may alleviate these practices and reduce associated foetal losses.

The highest overall foetal wastage, specifically for cattle and goats, was observed during the dry season of 2010. An earlier report on foetal calf wastage at the MMA showed a similar trend in 2009 (Alhaji 2011). It should be kept in mind that seasonal slaughter patterns and trends may be affected to varying extents by circumstances such as occasions and festivities with no fixed annual dates (e.g. Eid), salary increases and improved living standards, as well as uncertainties and risks such as drought.

The model used to analyse small-ruminant foetal wastage at the Minna abattoir revealed multiple peaks per year across the seasons, as well as inconsistent trend influences. These fluctuations are not unexpected. Analysis of the dates of Eid el Kabir from 2001 to 2012 showed that foetal wastage peaked in the months leading up to the festival. Other studies have similarly associated higher sheep and goat slaughter, together with higher levels of lamb and kid foetal wastage, around festive periods (Alade, Sadisu & Gambo 2011; Bokko 2011; Garba *et al*. 2010; Tizhe *et al*. 2010). Observant Nigerians prepare for such festivals effectively through earlier purchase of small ruminants, predominantly sheep but also goats. During the festivities, these animals (primarily sheep) are slaughtered mostly in homesteads, and therefore the available data about sheep slaughter were relatively scant. Efforts should be directed at encouraging abattoir slaughter even during such festivities and special occasions.

Our results show that more goats were slaughtered than cattle or sheep at the Minna abattoir, although more foetal sheep losses were observed than for the other ruminants. This is similar to observations in other studies (Alade *et al*. 2011; Khan & Khan 1989; Mukasa-Mugerwa & Tekelye 1988). It is possible that the relatively small size and associated easier handling, ready availability, preference for taste and relatively lower costs may drive the preferential slaughter of small ruminants over large ruminants. This may apply to the slaughter of goats, but not necessarily for sheep. Seasons and festivities affect the slaughter of sheep and many home slaughters of this species occur around Eid.

Our study highlighted slaughter trends across ruminant livestock and confirmed seasonal patterns of foetal losses amongst pregnant ruminant species presented for slaughter at the MMA. The trend and seasonality will likely continue for the foreseeable future unless significant changes occur in animal management practices in the industry. Although legislation currently exists to prevent foetal wastage, adherence to such legislation is difficult to enforce. Although we have offered possible reasons for the observed patterns, future studies should use empirical evaluations to explain the current patterns and predict the associated economic cost to the nation. Increased purchasing power and urbanisation, together with a growing population, will likely result in a continuing rise in the demand for meat. The current trends and seasonality of foetal wastage at abattoirs are incompatible with meeting future challenges.

The mainly pastoralist and transhumant systems of ruminant management influence the marketing structure for livestock in Nigeria. Herds are moved regularly in response to seasonal changes, the quality of grazing and the challenge posed by tsetse flies (southward in search of pastures and returned northward during tsetse fly infestation in the southern areas), and marketing systems closely follow this pattern. During these movements, sick and healthy animals are sold in open livestock markets along the routes, with live goats and sheep bought more regularly than cattle owing to the substantial price differences between small and large ruminants (Lawal-Adebowale 2012). Whether this pattern influenced the pattern of animal slaughters at the abattoir could not be confirmed in this study. It may be possible that peak slaughter occurs when more animals transit through the Minna area and an independent evaluation elsewhere may present a different result.

Our evaluation is subject to some limitations. First, the data were inconsistent for some years, with many missing values. This may have introduced bias and shifted the observed trends and seasonality. Future planning at the abattoir should consider targeted and detailed data sets to minimise such error. Second, the dynamics of domestic ruminant supply observed for the studied abattoir may not be representative of all Nigerian abattoirs and a similar study elsewhere in Nigeria might reveal a different outcome. Finally, we cannot confirm whether censorship (by butchers who want to use or sell foetal products and avoid confiscation) affected the data used for this study. Because the abattoir staff change often and there is no standardised format for reporting the slaughters, the possible effect of variation in data entry could not be determined. A standardised format for reporting is encouraged for future use at the abattoir.

## Conclusion

This empirical trend analysis of ruminant foetal wastage at the MMA demonstrated that seasonality is a major factor in determining slaughter patterns of pregnant ruminants. This slaughter pattern is likely to continue. We recommend a change in the current animal management practices to and enforcement of laws guiding against the indiscriminate slaughter of pregnant ruminants at Nigerian abattoirs. Future studies need to explore the use of empirical evaluations in analysing the current pattern and quantify the economic losses associated with abattoir foetal wastages.

## Figures and Tables

**FIGURE 1-A2:**
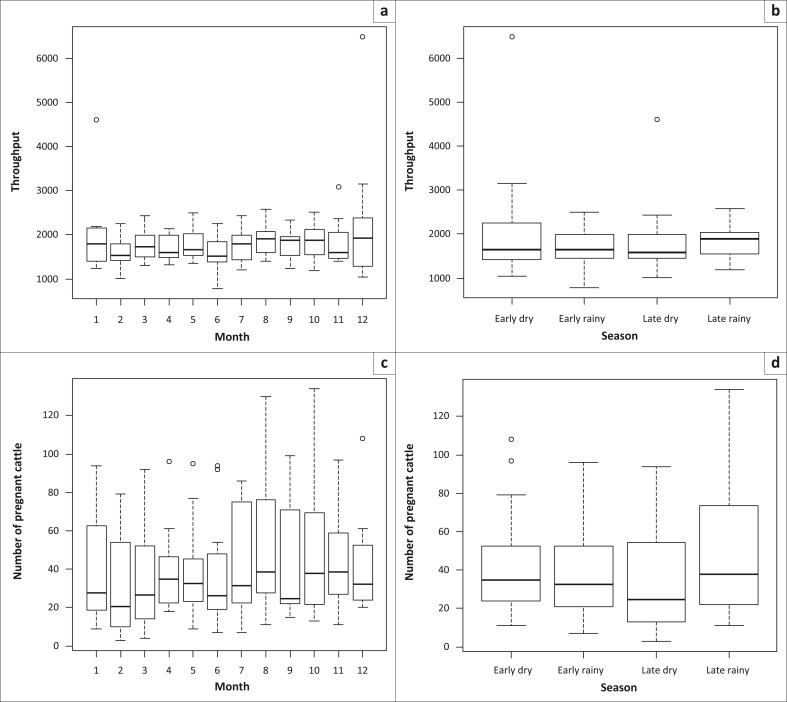

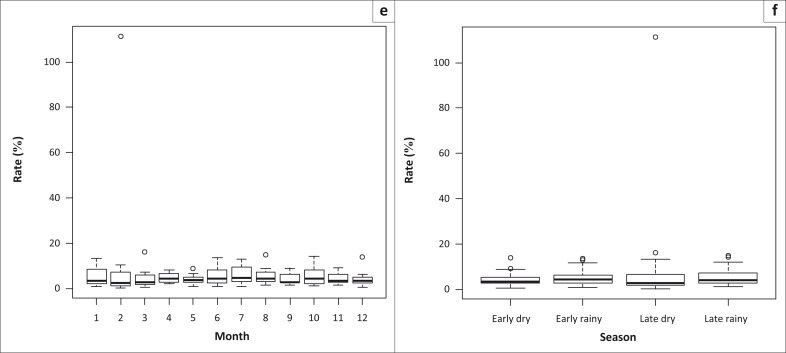
Monthly and seasonal rate of throughput, slaughter of pregnant animals and foetal wastage for cattle, Minna Metropolitan Abattoir, Nigeria, during 2001–2012. (a) Monthly throughput for cattle, (b) seasonal throughput for cattle, (c) monthly slaughter of pregnant cattle, (d) seasonal slaughter of pregnant cattle, (e) monthly rate of foetal wastages in cattle and (f) seasonal rate of foetal wastages in cattle.

**FIGURE 2-A2:**
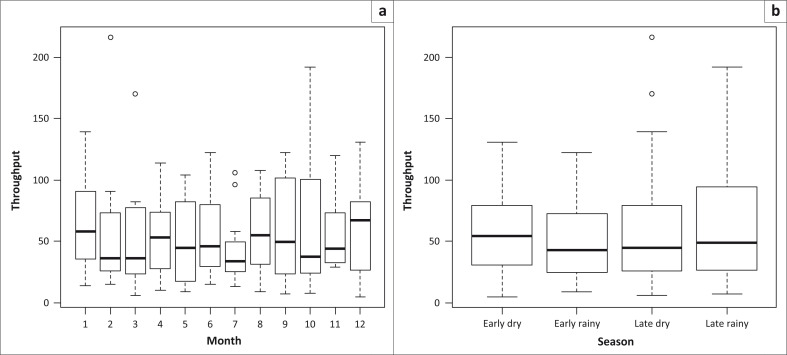

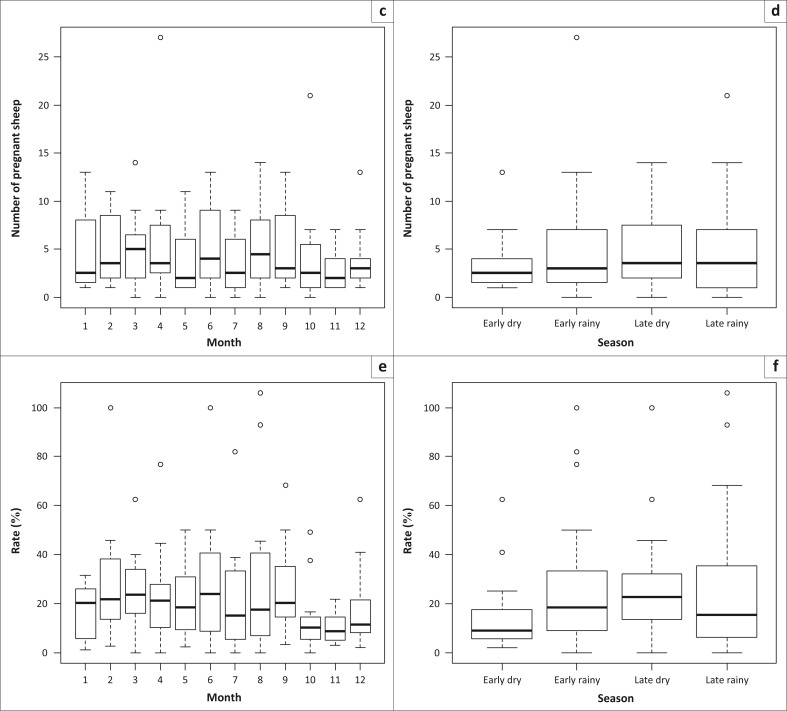
Monthly and seasonal rate of throughput, slaughter of pregnant animals and foetal wastage for sheep, Minna Metropolitan Abattoir, Nigeria, during 2001–2012. (a) Monthly throughput for sheep, (b) seasonal throughput for sheep, (c) monthly slaughter of pregnant sheep, (d) seasonal slaughter of pregnant sheep, (e) monthly rate of foetal wastages in sheep and (f) seasonal rate of foetal wastages in sheep.

**FIGURE 3-A2:**
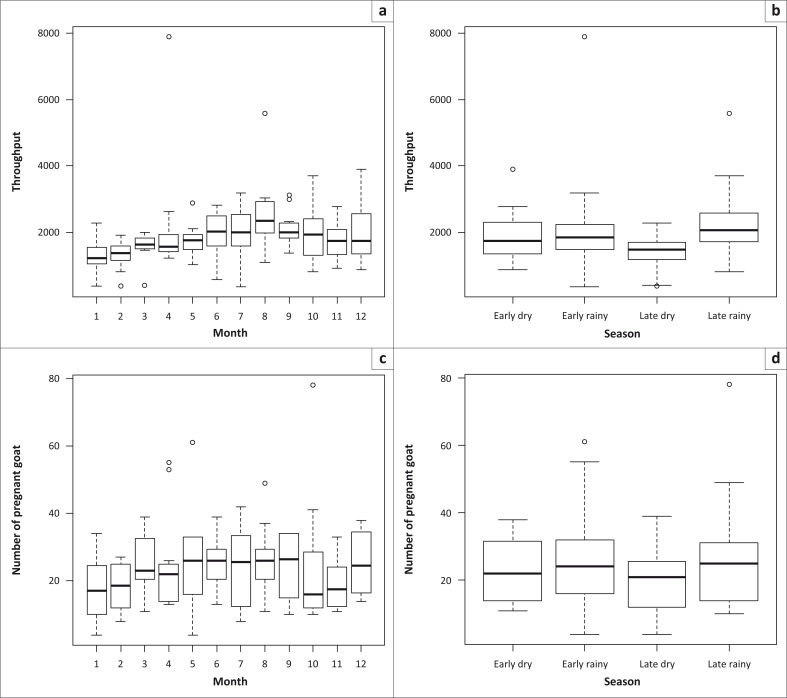

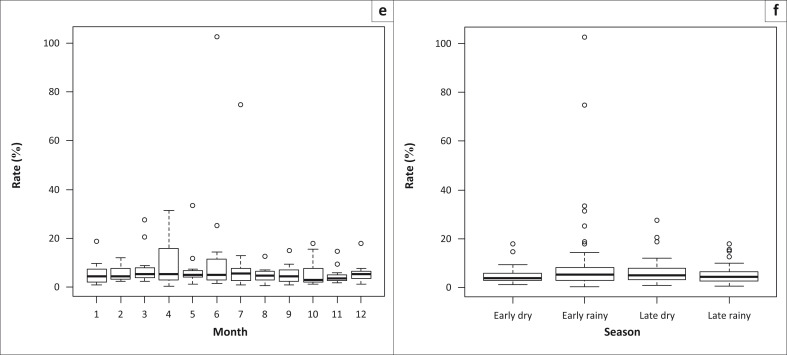
Monthly and seasonal rate of throughput, slaughter of pregnant animals and foetal wastage for goats, Minna Metropolitan Abattoir, Nigeria, during 2001–2012. (a) Monthly throughput for goat, (b) seasonal throughput for goat, (c) monthly slaughter of pregnant goat, (d) seasonal slaughter of pregnant goat, (e) monthly rate of foetal wastages in goat and (f) seasonal rate of foetal wastages in goat.

## Appendix 1

**TABLE 1-A1:** Case–control evaluation of monthly calf wastage using logistic regression analysis.

Month	Odds ratio	𝜒 ^2^	*P* > 𝜒 ^2^	95% confidence interval	95% confidence interval
1	1.000000	-	-	-	-
2	0.821084	8.21	0.0042	0.717373	0.9399788
3	0.847241	6.14	0.0132	0.743035	0.966962
4	1.001873	0.00	0.9770	0.882370	1.137560
5	0.880941	3.79	0.0515	0.775366	1.000893
6	1.076618	1.28	0.2575	0.947454	1.223389
7	1.172047	6.33	0.0118	1.035618	1.326447
8	1.165909	6.48	0.0109	1.035812	1.312346
9	0.972456	0.19	0.6589	0.859041	1.100844
10	1.163167	6.14	0.0132	1.032007	1.310997
11	1.052771	0.64	0.4220	0.928556	1.193603
12	0.645536	48.22	0.0000	0.569952	0.731143

Test of homogeneity (equal odds): 𝜒 ^2^(11) = 167.75.

Pr > 𝜒 ^2^ = 0.0000.

Score test for trend of odds: 𝜒 ^2^(1) = 0.56.

Pr > 𝜒 ^2^ = 0.4533.

**TABLE 2-A1:** Case–control evaluation of monthly lamb wastage using logistic regression analysis.

Month	Odds ratio	𝜒 ^2^	*P* > 𝜒 ^2^	95% confidence interval	95% confidence interval
1	1.000000	-	-	-	-
2	2.001005	9.26	0.0023	1.268680	3.156052
3	2.055609	9.88	0.0017	1.298865	3.253248
4	2.580671	18.46	0.0000	1.647478	4.042460
5	1.822541	6.14	0.0132	1.125526	2.951202
6	1.952434	8.72	0.0032	1.241798	3.069743
7	1.957314	7.38	0.0066	1.194541	3.207153
8	2.354098	14.41	0.0001	1.492653	3.712704
9	2.063984	10.18	0.0014	1.309479	3.253225
10	1.550559	3.45	0.0633	0.972447	2.472353
11	1.458941	2.69	0.1012	0.926216	2.298068
12	1.161412	0.39	0.5326	0.725588	1.859015

Test of homogeneity (equal odds): 𝜒 ^2^(11) = 36.78.

Pr > 𝜒 ^2^ = 0.0001.

Score test for trend of odds: 𝜒 ^2^(1) = 2.50.

Pr > 𝜒 ^2^ = 0.1141.

**TABLE 3-A1:** Case–control evaluation of monthly kid wastage using logistic regression analysis.

Month	Odds ratio	𝜒 ^2^	*P* > 𝜒 ^2^	95% confidence interval	95% confidence interval
1	1.000000	-	-	-	-
2	1.257524	5.80	0.0160	1.043143	1.515964
3	1.423937	15.92	0.0001	1.195922	1.695424
4	0.793240	6.90	0.0086	0.667110	0.943217
5	1.367343	12.68	0.0004	1.150217	1.625456
6	1.283522	8.04	0.0046	1.079691	1.525834
7	1.121650	1.66	0.1977	0.941782	1.335869
8	0.961567	0.20	0.6551	0.809638	1.142007
9	1.011726	0.02	0.8958	0.849702	1.204645
10	1.142256	2.23	0.1356	0.959061	1.360444
11	1.024481	0.06	0.8033	0.846970	1.239194
12	1.045261	0.25	0.6153	0.879509	1.242251

Test of homogeneity (equal odds): 𝜒 ^2^(11) = 88.17.

Pr > 𝜒 ^2^ = 0.0000.

Score test for trend of odds: 𝜒 ^2^(1) = 2.25.

Pr > 𝜒 ^2^ = 0.1338.
